# Cancer Metabolism and Ischemia-Reperfusion Injury: Two Sides of the Same Coin

**DOI:** 10.3390/jcm11175096

**Published:** 2022-08-30

**Authors:** Denise V. Nemeth, Enke Baldini, Salvatore Sorrenti, Vito D’Andrea, Maria Irene Bellini

**Affiliations:** 1 School of Osteopathic Medicine, University of the Incarnate Word, San Antonio, TX 78235, USA; 2 Department of Surgical Sciences, Sapienza University of Rome, 00185 Rome, Italy

**Keywords:** cancer cell metabolism, ischemia-reperfusion injury, hallmarks of cancer, aerobic glycolysis, angiogenesis, apoptosis, tumorigenesis

## Abstract

Cancer cells are characterized by the reprogramming of certain cell metabolisms via activation of definite pathways and regulation of gene signaling. Ischemia-reperfusion injury (IRI) is characterized by tissue damage and death following a lack of perfusion and oxygenation. It is most commonly seen in the setting of organ transplantation. Interestingly, the microenvironments seen in cancer and ischemic tissues are quite similar, especially due to the hypoxic state that occurs in both. As a consequence, there is genetic signaling involved in response to IRI that has common pathways with cancer. Some of these changes are seen across the board with many cancer cells and are known as Hallmarks of Cancer, among which are aerobic glycolysis and the induction of angiogenesis. This literature review aims to compare the metabolic pathways that are altered in cancer tissues and in normal tissues subjected to IRI in order to find common adaptive processes and to identify key pathways that could represent a therapeutic target in both pathologies. By increasing our understanding of this relationship, clinical correlations can be made and applied practically to improve outcomes of transplanted organs, given the known association with acute rejection, delayed graft function, and poor graft survival. The following metabolic pathways are discussed in our review, both in the setting of cancer and IRI: apoptosis, glycolysis, and angiogenesis. The role of the immune system in both pathologies as well as mitochondrial function and the production of reactive oxygen species (ROS) are reviewed.

## 1. Introduction

It is known that some metabolic pathways are activated in ischemia-reperfusion injury (IRI) as well as in cancer [1,2]. This is because the microenvironments seen in cancer and ischemic tissues are rather similar, especially due to the hypoxic state that occurs in both [3,4]. Hypoxia is generically defined as a condition of oxygen insufficiency, either acute, because of microcirculation injury and hypoperfusion, or chronic, due to permanent vascular shortage, unresolved tissue edema, and inflammation. Physiological normoxia, also named physioxia, is the normal oxygenation level in peripheral tissues and is significantly lower than that present in the air (21% of oxygen), ranging between 2% and 14% of oxygen depending on the individual tissue [5]. Thus, the condition of physiological hypoxia does not refer to a precise threshold value but is the level of oxygenation to which the tissues will respond to maintain their preferred oxygen level. If a tissue fails to return to the state of physioxia, the persistence of a low oxygen level will cause tissue necrosis and consequent functional impairment. However, tissue response varies depending on the cell type and the onset of hypoxia in healthy or pathologic tissues [6]. Certain tissue types are quickly and severely damaged by low oxygen levels; others can withstand some degree of hypoxia, even prolonged, while cancer tissues can develop a subpopulation of cells that adapt to hypoxic conditions and acquire new malignant features [7].

This literature review aims to compare the metabolic pathways that are altered in cancer tissues and in normal tissues subjected to IRI in order to find common adaptive processes and to identify key pathways that could represent a therapeutic target in both pathologies. By increasing our understanding of this relationship, clinical correlations can be made and applied practically to improve outcomes of transplanted organs, given the known association with acute rejection, delayed graft function, and poor graft survival. 

**Figure 1:**
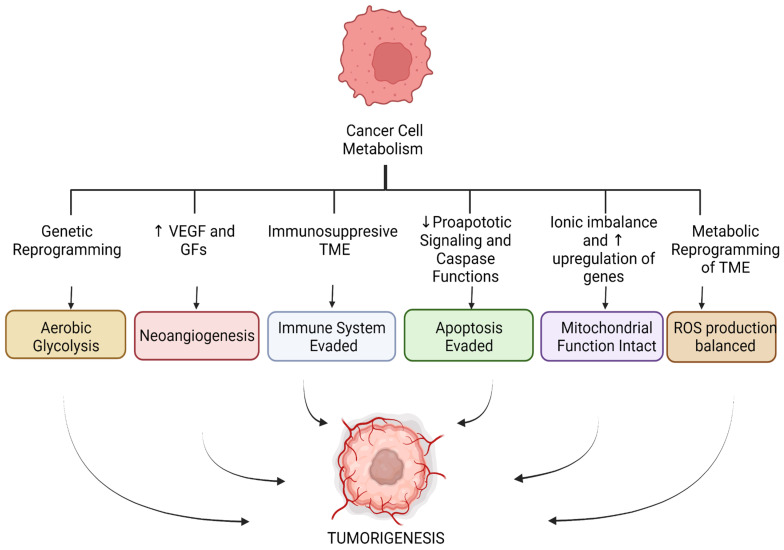
Metabolic pathways altered in cancer cell metabolism. (VEGF = vascular endothelial growth factor, GFs = growth factors, TME = tumor microenvironment.)

**Figure 2:**
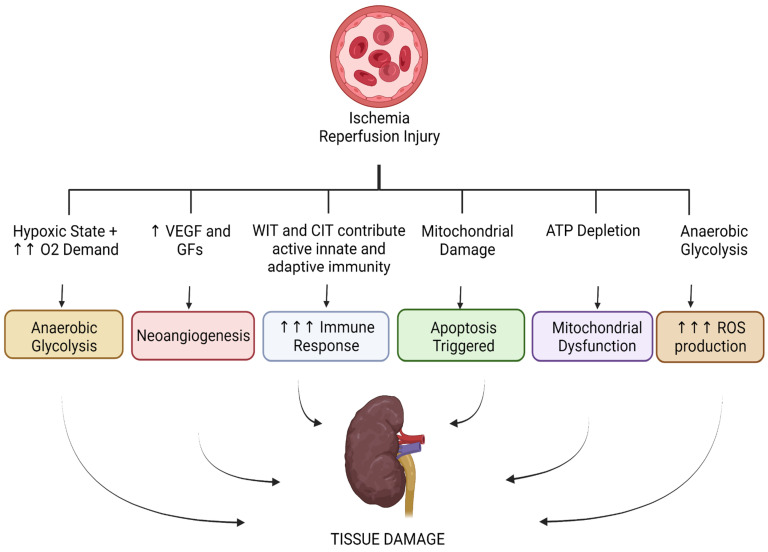
Metabolic pathways altered in ischemia–reperfusion injury. (VEGF = vascular rndothelial growth factor, GFs = growth factors, WIT = warm ischemia time, CIT = cold ischemia time.)

The following metabolic pathways are discussed in IRI and cancer: apoptosis, glycolysis, and angiogenesis. The role of the immune system in both pathologies, as well as mitochondrial function and the production of reactive oxygen species (ROS), are also addressed. These pathways are illustrated in Figure 1 and Figure 2 below. 

## 2. Literature Search

A review of the literature was performed using the following databases and tools, respectively: PubMed and the Primo Ex Libris tool. A total of 117 articles and studies were reviewed. These references all discussed, in IRI and in cancer, the Warburg effect, genetic pathways up/downregulated, mitochondria/mitochondrial function, glycolysis, apoptosis and necrosis, and angiogenesis. The following keywords were used for search purposes: “cancer”, “tumor”, “cancer cell metabolism”, “ischemia reperfusion injury”, “hallmarks of cancer”, “warburg effect”, “aerobic glycolysis”, “glycolysis”, “angiogenesis”, “neoangiogenesis”, “apoptosis”, “tumorigenesis”, “HIF”, “Hypoxia Inducible Factor”, “tumor microenvironment”, “microenvironment”, “immunity”, “stroma”, and “hypoxia”. Textbooks and E-books reviewed included Critical Care Nephrology (Third Edition), Clinical Biochemistry: Metabolic and Clinical Aspects (Third Edition), A Cell: A Molecular Approach (Second Edition), and StatPearls. We excluded papers that were not in English and those which were solely published as abstracts in conferences. 

### 2.1. Tumor Microenvironment and the Significance of Stroma in Cancer and IRI

Tumorigenesis has been described as the loss of original cell features and the gain of malignant properties in normal cells. Some of the typical changes in cancer cells include evasion of apoptosis, sustained proliferation, insensitivity to anti-growth signals, altered glucose metabolism leading to anaerobic glycolysis, and induction of angiogenesis. These acquired and altered functional capabilities are better known as the Hallmarks of Cancer [4,8] and occur in response to an increase in demand for nutrients and oxygen that cancer needs to spread and metastasize [9]. Tumors, particularly those at high rates of proliferation, develop a hypoxic core due to the demand for oxygen becoming higher than the supply [7]. The cellular environment in which cancer cells reside and grow is commonly referred to as the tumor microenvironment (TME) [10]. It is essentially a heterogeneous framework that includes extracellular matrix (ECM), immune cells, cancer-associated fibroblasts (CAFs), endothelial cells, pericytes, and mesenchymal stem cells. The TME is important because it is the site of complex processes that facilitate not only tumorigenesis but also angiogenesis, invasion, and metastasis, all of which are key to the successful proliferation of cancer cells [11]. In many instances, the literature describes the TME as a highly efficient ecosystem able to evolve simultaneously with the expansion of the cancerous mass [1,2]. Its reshaping relies heavily on the recruitment of distant immune cells that will acquire the role of either tumor-promoting cells or tumor-suppressing cells [12]. Because these cells are typically recruited only under a condition of injury or noxious stimulus, the TME must be under a constant state of stress. This requirement is met by the sum of the incessant demands of oxygenation leading to hypoxia, increased levels of ROS, a chronic level of inflammation, low pH, nutrient deprivation, and genomic instabilities leading to heterogeneity in the TME itself [13]. As part of the TME, the tumor stroma can have a significant impact on the metabolic changes observed in cancer. The stroma consists of connective tissue that functions as a support for an organ, and likewise, it represents a scaffold in which the tumor mass can be nurtured and thrive. Moreover, the tumor-stroma crosstalk influences in a relevant way tumorigenesis and cancer progression, as well as resistance to anticancer therapeutics [3]. The stroma adjacent to the tumor has several components, of which the main ones are the ECM and specialized connective tissue cells, such as cancer-associated fibroblasts (CAFs) and mesenchymal cells [14]. CAFs are activated fibroblasts secreting different kinds of cytokines, chemokines, and growth factors and enabling cell-to-cell interactions that yield processes beneficial to tumor progression [15]. CAFs remodel ECM giving rise to desmoplasia and fibrosis, which limit the access of drugs and immune cells to TME and allow for crosstalk between immune cells whose final outcome can be immunosuppression [16]. The means which contribute to the activation of CAFs are many and include the presence of ROS, inflammatory cytokines, DNA damage secondary to chemoradiation and growth factors (GF) such as the platelet-derived (PDGF) and the fibroblast (FGF) ones [17]. The set of these interconnected actions of stroma and tumor encourages invasion and metastasis and is also likely to prompt resistance to therapeutic drugs and tumor relapse. On the other hand, it could also represent a good target for chemotherapy agents [3].

When it comes to IRI, the stroma is of equal significance. Recent work has shown that ischemia causes the tissue microenvironment to become acidic. This change in pH has immunologic consequences, such as the suppression of T regulatory cell generation, which can further lead to aggravation of IRI [18]. An acidic microenvironment can also lead to an increase in neutrophil recruitment, downregulation of protein, DNA and cAMP synthesis, and upregulation of nitric oxide synthase in macrophages. During the process of IRI, there is also crosstalk between the immune system and metabolic components that will mediate both the inflammatory response and the extent of the injury [19]. These changes in the microenvironment are of significance because they can be targets in the treatment of IRI. As we discuss further, IRI has multiple pathways that lead to injury, which can then result in decreased graft function, organ failure, and an overall decrease in long-term survival. IRI is a process that can produce an increased amount of ROS and inflammatory components leading to vascular permeability, edema, and damage to the endothelium. When homeostasis is disrupted, intracellular ion balances will change, leading to massive inflammation and damage, including oxidative stress and even mechanical lysis. The relationship between IRI and the stroma is of such significance that we now pursue strategies in an attempt to attenuate the detrimental effects of this process, such as organ preservation solutions (OPS), dynamic preservation and especially ex vivo normothermic perfusion (EVNP) to resemble a physiological environment. It has been, in fact, demonstrated that dynamic perfusion significantly reduces the incidence of delayed graft function and similar postoperative complications [20]. OPS represents a major driver of IRI in organ reconditioning. These OPS are synthetic, sterile solutions that are used to minimize the damage caused by IRI [21]. EVNP involves the use of a perfusion solution that mimics the physiological environment to rewarm the graft at hand to a normothermic temperature range. This restores the metabolic processes and allows for the assessment of graft quality assessment prior to transplantation [22,23]. These solutions are not able to fully mitigate the effects of IRI but are able to reduce them significantly. Research regarding the appropriate composition and protocols for the administration of these perfusates is still underway, but many institutions use perfusates that are red-blood-cell-based and nutrient enriched for a period of 60 min [24].

### 2.2. Glycolytic Pathways in Cancer and IRI

**Figure 3:**
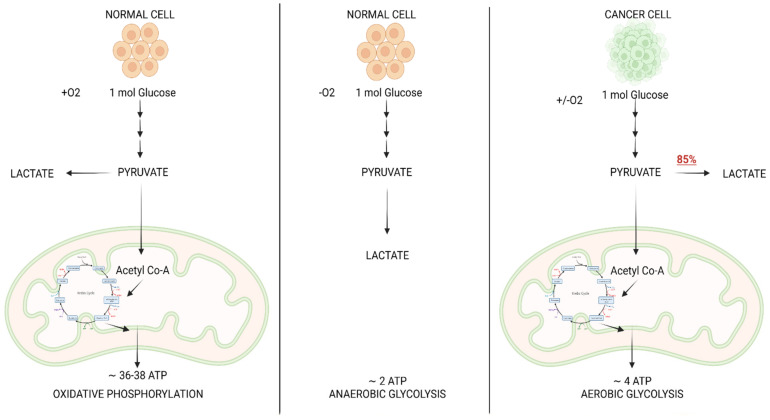
Comparison of metabolic pathway of glucose in normal cell vs. cancer cell.

Glycolysis is a sequence of events that results in the breakdown of one molecule of glucose into two molecules of pyruvate with the ultimate goal of supplying the cell with energy, or ATP [25]. The pyruvate can then take one of two paths, the first of which requires oxygen, while the second is typically initiated in the absence of oxygen and is best known as anaerobic glycolysis. In the presence of oxygen, pyruvate is converted to Acetyl CoA by the enzyme pyruvate dehydrogenase [26]. The Acetyl CoA can then enter the Krebs Cycle, which takes place in the mitochondrial matrix and produces NADH and FADH2. The chemical energy contained in NADH and FADH2 is used to make adenosine triphosphate (ATP) via a series of redox reactions and the formation of a proton gradient through the inner mitochondrial membrane, in a process called oxidative phosphorylation. Overall, glycolysis, Krebs cycle, and oxidative phosphorylation yield an approximate 36–38 ATP per glucose molecule (Figure 3), which can be used as an energy source for cells. In anaerobic glycolysis, glucose is converted to lactate by the enzyme lactate dehydrogenase, which concurrently oxidizes NADH to NAD+ [27]. This process is much less efficient in supplying energy because it produces only two molecules of ATP. In a normal cell, this pathway typically takes place as a response to tissue hypoxia. However, in cancer, a distinctive cellular metabolic alteration occurs, referred to as aerobic glycolysis, also known as the Warburg Effect [28]. This is a phenomenon in which cancer cells opt to undergo the glycolytic pathway and metabolize glucose into lactate to sustain energy demands, regardless of the presence of oxygen [13].

A widely held misconception arising from Warburg’s initial hypothesis is that cancer cells use the glycolytic pathway in the presence of oxygen due to defective mitochondria as a way to obtain ATP. However, there is evidence that in most tumors, glycolysis is upregulated, and their mitochondrial function is not impaired. Therefore, oxidative phosphorylation continues as in normal tissues [29]. To date, it is believed that cancer cells opt to go through the glycolytic pathway to support tumorigenesis and produce increases in biomass, as well as to support proliferation and overall progression. Hence, one of the major functions of this pathway is to maintain elevated levels of intermediates of the glycolytic pathway in order to fuel anabolic reactions [30].

The ability of cancer cells to undergo aerobic glycolysis lies in the reprogramming of glucose metabolism via activation of oncogenes, inactivation of tumor suppressors, and alterations to transcription factors. One of the most commonly altered pathways in cancer is that of PI3K/AKT/mTOR. The PI3K consists of a large family of lipid kinases that phosphorylate -OH groups of phosphatidylinositols on the inner side of the plasma membrane [31]. PI3K signaling pathways play an important role in processes such as proliferation, inflammation, cell survival and death, metabolism, and cancer progression [32]. PI3K specifically supports anabolic processes via the uptake of glucose and essential amino acids. Its unique characteristic is the ability to regulate glucose transporter expression (GLUT1), enhance glucose capture, and stimulate phosphofructokinase activity. These processes are of utmost importance to tumorigenesis, and in fact, PI3K is commonly seen upregulated in cancer [33]. Constitutive activation of the PI3K signaling can occur due to several mechanisms, the best known of which includes inactivating mutations of negative regulators such as PTEN (phosphatase and tensin homolog), TSC1, and TSC2 (TSC- tuberous sclerosis complex) [34].

PI3K starts a signaling cascade that activates Akt, which in turn modulates several downstream molecules, including mTOR (mammalian target of rapamycin) [35]. Research has previously shown that increased levels of Akt phosphorylation directly correlate with increased rates of glucose metabolism in cancer cells. In a non-pathological state, mTOR heavily regulates cell growth and division. Nevertheless, in malignant tumor cells, an abnormally phosphorylated mTOR promotes uncontrolled tumor cell growth, invasion of nearby tissues, and metastasis [36]. mTOR is activated when there is an ample supply of nutrients, which can further support anabolic activities as well as the storage and use of energy. Rather, when nutrients are in scarcity, the body will inhibit the activation of mTOR to preserve energy stores. Some literature goes as far as calling mTOR the master stimulator of cell growth [20].

Tumor cells require large amounts of macromolecules in order to meet the demands for growth and division. Abnormal activity of the mTOR pathway often occurs in tumors, where mTOR plays a core role in regulating metabolism by inducing new lipid synthesis and increasing the absorption of nutrients [18].

In addition, mTORC1, one of the mTOR complexes, was shown to enhance the expression of the transcription factors hypoxia-inducible factor 1-α (HIF-1α) and MYC, which in turn stimulate the transcription of several genes involved in glucose transport, glycolysis, ribosome biogenesis and mitochondrial function [37].

HIFs (hypoxia-inducible factors) are frequently overexpressed in cancer cells independently from a hypoxic environment. HIF1α mRNA was found elevated in pre-neoplastic breast, colon, and prostate lesions and remained elevated when cells were cultured in normoxic conditions, which further suggests that hypoxia is not necessary for HIF-1α activation in cancer cells [22]. The constitutive expression of both HIF-1α and HIF-2α can be due to different causes, that is overstimulation of the PI3K/Akt/mTOR pathway, mutations in oncogenes that affect the HIF gene transcription rate (e.g., p53, Bcl2, Myc, and Ras), or functional loss of the Von Hippel-Lindau (VHL) protein, responsible for HIF-1α degradation [38].

In particular, HIF-1α is important in determining the way in which cells will utilize glucose, switching metabolism towards aerobic glycolysis. In fact, HIF-1α transcriptional targets include genes encoding for the glucose membrane transporters GLUT1 and GLUT3, and also glycolytic enzymes such as hexokinases and phosphoglycerate kinase 1. In addition, HIF-1α increases the expression of the pyruvate dehydrogenase kinase, which in turn inactivates the pyruvate dehydrogenase. Hence, pyruvate conversion to acetyl-CoA is prevented, and the Krebs cycle is inhibited [17]. These combined actions will yield both increased glucose utilization and lactate production [20].

The constitutive expression of HIF1α and HIF2α also confers cancer cells’ resistance to apoptosis [39]. Therefore, stabilization of HIF-1α is associated with poor prognosis and higher rates of mortality in different cancers [40].

Overall, as a result of the shunting of glucose into aerobic glycolysis, with augmented production of lactate, downstream pro-tumorigenic effects arise in the form of increased angiogenesis, collagen remodeling, autophagy, decreased T cell activity and decreased leukocytes [41]. Historically, the Warburg effect has equated to cancer cell metabolism. At present, it is understood that cancer cell metabolism is more than a reprogramming of a single cellular metabolic pathway and is essential for the development of malignancy.

In IRI we see a hypoxic state, which, coupled with a high demand for oxygen, will trigger a sequence of events that culminates in a high rate of anaerobic glycolysis. This differs from the aerobic glycolysis that we see in cancer cell metabolism in that there is truly no oxygen supply present. However, the outcomes are the same as those seen in aerobic glycolysis because the glycolytic pathway is identical. This produces increased levels of ROS, causing oxidative stress, damage, and ultimately apoptosis [42]. Therefore, current trends in organ transplantation are now to add oxygen during machine perfusion in an attempt to make a metabolic switch from anaerobic glycolysis to aerobic glycolysis. This has shown promising results in terms of postoperative complications [43,44], as this shift in metabolism has been associated with decreased ROS production and protection from injury and apoptosis in IRI [45].

### 2.3. Neoangiogenesis in Cancer and IRI

**Figure 4:**
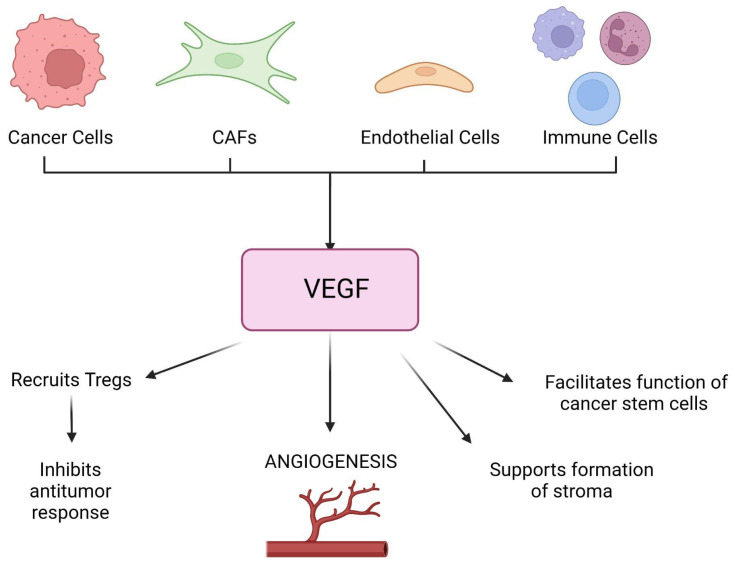
VEGF secretion and its effects in malignant tumors. (CAF = cancer-associated fibroblasts, VEGF = vascular endothelial growth factor, Tregs = T-regulatory cells.)

Another cellular event commonly seen in cancer cells is the induction of angiogenesis, also considered an additional Hallmark of Cancer. Neo-angiogenesis is a critical process that is needed to sustain tumor growth, survival, and proliferation. It also plays a role in metastasis and invasion [46]. Cancer cells need nutrients and oxygen in order to successfully continue undergoing proliferation. The anabolic processes by which tumors gain biomass and increase their size require an established vascular network that can provide not only nutrients and oxygen but also dispose of waste and carbon dioxide [47]. These needs are met through neo-angiogenesis, a process that arises very early during the development of invasive cancers (Figure 4). [48] As a tumor progresses, there is what some call an angiogenic switch, which is essentially a condition in which proangiogenic signals and the relative receptors are constitutively activated and/or upregulated [49]. This alteration is due to either continuous stimulation by an excess of ligands secreted in the TME or activating mutations of the receptor itself [37].

There are many signaling molecules involved in the process of angiogenesis, some inhibitory in nature and others stimulatory. Some signaling molecules worthy of note include vascular endothelial growth factor (VEGF), epidermal growth factor (EGF), transforming growth factor beta (TGF-β), fibroblast growth factor (FGF), thrombospondin-1 (TSP-1), and platelet-derived growth factor (PDGF), to name a few [50]. The up- or downregulation of each of these molecules yields different results depending on the final balance between pro- and antiangiogenic factors. Thus, while some tumors exhibit high levels of angiogenesis, others are hypovascularized. Amongst the stimulatory molecules, VEGF, PDGF, EGF, FGF, and TGF are those of most significance.

The VEGF protein is an endothelial cell-specific mitogen that promotes vascular hyperpermeability and the formation of new blood vessels. Hypoxia is one of its major regulators via HIF [51]. In tumors, VEGF can be secreted by malignant cells, CAFs, endothelial cells, and immune cells. Besides the induction of angiogenesis, it supports the formation of desmoplastic stroma, promotes dedifferentiation and epithelial-mesenchymal transition, facilitates the function of cancer stem cells, and also acts as a chemoattractant to recruit regulatory T (TReg) cells that inhibit antitumor immune responses [52]. PDGF regulates cell growth and division [53]. Not only is it a powerful activator of mesenchymal stem cells, but it also stimulates chemotaxis and proliferation, which aids greatly in tissue repair [54]. In combination with VEGF and FGF, it has been associated with the vascularization of malignant tumors. Aberrant PDGF-PDGFR signaling leads to tumor angiogenesis by recruiting pericytes to vessels, contributing to the secretion of proangiogenic factors, stimulating endothelial cell proliferation, migration, sprouting, and tube formation, and promoting lymphangiogenesis as well as lymphatic metastasis [55].

Alterations leading to upregulation of the FGF-FGFR signaling pathway are also paramount in oncogenesis, participating in the regulation of proliferation, differentiation, cell survival, immune evasion, and neoangiogenesis [56]. Similarly, hyperactivation of the EGF-EGFR signaling pathway stimulates tumor growth, invasion, and metastatic activity and increases the production of VEGF and FGF in a great variety of tumor cells. 

TGF-β is a multifunctional cytokine with a prominent role in regulating cell growth, differentiation, apoptosis, motility, immunity, ECM production, and angiogenesis. It has a paradoxical role in cancer, as in earlier stages, it inhibits cellular transformation and prevents cancer progression, essentially acting as a tumor suppressor. However, in later stages, it promotes tumor progression via immunosuppression, mesenchymal transition, and angiogenesis. In like manner, TGF-β acts on endothelial cells in opposite ways depending upon the cellular context of tumor cells, epithelial cells, and TME [57]. In some cancers, it can increase microvessel density by inducing key angiogenic factors such as VEGF, while in others, it exerts an angiostatic effect through the induction of TSP-1, a potent angiogenic inhibitor. TSP-1, on the other hand, opposes the effects of all the previously mentioned molecules. It is a protein found in the ECM that has antiangiogenic effects and inhibits endothelial migration and proliferation [58].

It is important to note that in healthy tissues and under normal conditions, new blood vessels are laid out in an organized fashion. This is regulated by the same aforementioned molecules involved in cancer-related angiogenesis. In contrast to that, the blood vessels created in neoplastic processes are structurally abnormal [59,60]. They exhibit immature features and are characterized by excessive branching, tortuous and enlarged vessels, erratic blood flow, micro hemorrhaging, and leakiness [60,61]. Pericytes, which are contractile cells present at intervals along the walls of capillaries, are a key player in the formation of these aberrant vessels [62]. Recent studies have shown that the interaction between pericytes and malignant cells causes modifications in the TME that favor tumor growth. The structural abnormalities of tumor vasculature contribute to differences in perfusion throughout the tumor, which results in intratumoral heterogeneity [43]. This is of importance in the treatment of targeted and immune therapies, as the response inside the tumor mass will likely not be uniform and can lead to therapy resistance and failure [63].

An additional contributor to tumor neoangiogenesis is the previously discussed increase in lactate production via aerobic glycolysis. The export of lactate outside of the cell is facilitated via upregulated Monocarboxylate Transporters 1–4 (MCT 1–4). This, in turn, increases VEGF protein expression, leading to angiogenesis and tumor growth. [41] 

**Figure 5:**
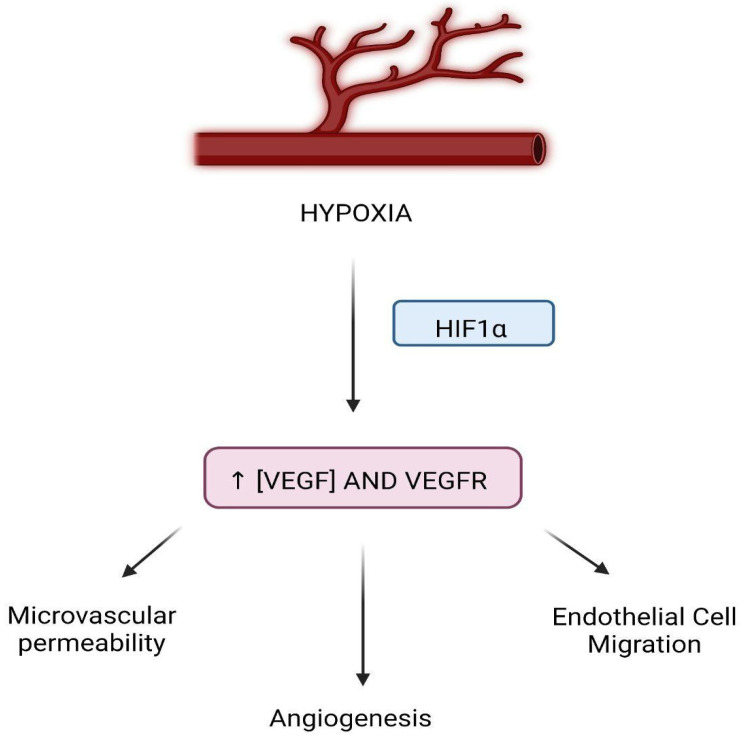
VEGF in the setting of IRI. (HIF = hypoxia-inducible factor, VEGF = vascular endothelial growth factor, VEGFR = vascular endothelial growth factor receptors.).

Reperfusion of an ischemic organ is absolutely necessary for the recovery of its full function. Nevertheless, when said organ is reperfused rapidly, this can cause additional injury in the form of IRI. IRI damages both the macro and microcirculation, particularly in the setting of organ transplantation. Hypoxia will stimulate higher levels of VEGF to be expressed during a state of ischemia via some inducible factors [51] (Figure 5). Despite research efforts, the evidence regarding the effects of IRI in humans, specifically in relation to VEGF levels in IRI patients, has not reached a universal consensus. While some studies showed an increase in VEGF as a consequence of IRI in comparison to healthy individuals, even when adjusting to confounding factors [64], others report the opposite effect. VEGF is well known to contribute to vasculogenesis [65], as it plays a crucial role in the recruitment of endothelial progenitor cells, which have the capability of aiding in tissue repair and organ function restoration [66]. VEGF and VEGF receptors (VEGFR), respectively, can be expressed in different areas of the organ at hand. For example, in the kidney, VEGF is predominantly expressed in tubular epithelial cells, and VEGFR are mostly seen in cells of the preglomerular, glomerular and peritubular areas of the kidney. [67] Additionally, because it simultaneously increases microvascular permeability and contributes to endothelial cell migration, it has the potential to reduce IRI [68]. 

### 2.4. The Immune System in Cancer and IRI

Malignancy activates immune responses within the body and induces many functional and compositional changes to the immune system [46]. Both the innate and the adaptive immune systems play an important role in cancer progression by establishing a complex network of interactions with each other and also with cancer cells and TME. Basically, immune cells can be distinguished into tumor-antagonizing cells and tumor-promoting cells. The former includes effector T lymphocytes, natural killer cells, dendritic cells, proinflammatory macrophages, and neutrophils; the latter mainly consists of regulatory T cells (Tregs) and myeloid-derived suppressor cells [69].

Initially, natural killer cells play a role in the identification and elimination of immunogenic cancer cells. As cancer grows, other components of the innate immune system intervene and eventually trigger the activation of the adaptive immune system. Tumor-associated macrophages (TAMs) and neutrophils (TANs) are no less important in the immune response to cancer. They secrete growth factors, cytokines, and proteolytic enzymes that favor cancer cell migration, ECM degradation, tumor vascularization, and epithelial-mesenchymal transition [70]. In addition, they influence the mobility and activity of dendritic cells, T helper cells, and effector T cells [49]. They are both implicated in the early stages of cancer development as well as in metastatic spreading and resistance to therapy. In several cancer types, high-grade TAMs and/or TANs correlate with poor prognosis and reduced overall survival [50,71]. Homing and the activity of innate immune cells can be boosted by the TME, further highlighting its importance in tumor progression. 

The sequence of events that lead to the death of malignant cells by means of the adaptive immune response is best known as the cancer-immunity cycle. The literature describes seven major steps in this cycle: (1) initial release of a neoantigen, which is captured by antigen-presenting cells (APCs); (2) APCs present the neoantigen to T cells via major histocompatibility complexes I and II (MHC I and II); (3) T cells are primed; (4) they migrate towards the tumor; (5) they infiltrate the tumor; (6) they recognize specifically cancer cells; (7) they annihilate cancer cells [72].

Overall, the immune response generated during the cancer-immunity cycle is relevant because the inflammatory response generated in the final steps is what allows for the killing of the tumor cells. 

Dendritic cells, which are APCs, are often referred to as a bridge between the innate and adaptive immune systems and are also important in the defense against cancer [73]. However, despite the potential that tumor-associated dendritic cells have to initiate an antitumor response, it is known that they are generally faulty and fail at achieving this function, ultimately contributing to immunosuppression in cancer [74]. First, neoantigens may not be identified by dendritic cells and T cells as non-self, resulting in the activation of T regulatory cells. Secondly, T cell homing or infiltration into the tumor may not occur properly, or TME may secrete immunosuppressive factors. Furthermore, many oncologic patients manifest a diversion of hematopoiesis towards an expansion of immature myeloid cells (i.e., dendritic cells, neutrophils, and monocytes), which penetrate the TME and contribute to local immunosuppression.

Overall, the role of stroma within cancer immunity is immunosuppressive. However, the immune system can still generate a response that, though not robust enough to destroy the primary tumor, can, in fact, prevent the metastasization and growth of a secondary tumor. This phenomenon is called concomitant immunity, and it is of note in metastatic disease [75]. Metastases are considered to be secondary tumors and are thought to not have the advantage of an associated stroma that has immunosuppressive features, hence their more vulnerable nature.

In IRI, the impairment caused at the microvascular level leads to further metabolic disorders and pH derangements in the transplanted organs. The reperfusion that follows will cause a calcium abnormality as well as an increase in free radicals that will then facilitate an immune response [76]. Therefore, it is this same robust immune response that leads to injury of host tissue and, subsequently, organ damage and death. This causes a paucity in the number of organs available for transplantation, as well as a marked increase in both long- and short-term negative outcomes for patients [77]. Efforts must be made to halt this process to prevent graft function loss. This link is of importance because understanding the immunosuppressive nature of cancer cells can lead to a better understanding of immunotherapy in the setting of IRI and vice versa.

Both the innate and adaptive immune systems have been recognized to play key roles in IRI during organ transplantation. Factors leading to delayed graft function (DGF) are of chief concern and are largely caused by the immune response previously mentioned. IRI is a main driver of graft dysfunction, as it can cause apoptosis of cells in the renal tubules and lead to DGF, which is an acute renal failure status post transplantation. DGF in itself leads to higher rates of allograft immunogenicity, which in turn results in acute organ rejection and can also lead to chronic allograft injury [76,78]. This becomes important when designing protocols to reduce the severity of the injuries that are associated with IRI and the immunological process innately involved with organ transplantation [79]. Several immune activation pathways have been recognized. Some of these pathways have opposing pro and anti-inflammatory functions that are determined by the stage of ischemia-reperfusion [80]. 

There are several time periods to consider that play a crucial role in IRI. These are warm ischemia time (WIT) and cold ischemia time (CIT), both of which have a strong correlation with DGF as well as the overall survival of the allografts [81], due to the considerable immune response that is mounted as a result of their presence [82].

WIT consists, in fact, of two separate time periods in the transplant process. The initial WIT is the time between the cessation of blood flow to the organ and its placement of it in a cold environment during procurement. It is a key difference between organs coming from donation after circulatory death (DCD) donors vs. those coming from the donation of brain dead (DBD) donors [83]. The second WIT takes place intraoperatively during the time of anastomosis of the graft. It begins with the removal of the procured organ from the cold storage and ends with the reperfusion of the allograft and occurs both in DBD and DCD. Cold ischemia time (CIT) is considered to be the time between when blood supply to an organ is discontinued and the organ is chilled and when the organ is removed from the cold preservation and rewarmed by initiating the anastomosis time. 

As with cancer cells, there is an immune response that is triggered, creating an inflammatory cascade. In the case of IRI, the process causes lysis which can then cause release of cellular contents. The components then become damage-associated molecular patterns (DAMPS) that can be recognized by toll-like receptors (TLRs) on immune cells [84,85]. This will then activate transcription factors that will further cause the release of inflammatory cytokines such as interferon (IFN)-α/β and IL-1, along with the production of multiple IRI aseptic inflammatory mediators [86]. 

Some research shows that macrophages are also important participants in the pathophysiology of IRI during organ transplantation. As resident immune cells become activated during IRI, they promote proinflammatory cytokines and chemotactic recruitment of other immune cells to the site of IRI, which helps in sustaining and multiplying the immunological response [87]. It seems that the influx of macrophages to the site of IRI contributes to injury of the organ via the secretion of cytokines, chemotaxis of neutrophils, and the initiation of apoptosis [74]. They are also able to sense DAMPs and produce IL1,6,8,12 and TNF-α [88]. Additionally, research shows that T-cell-mediated immunity is significant in the setting of IRI in organ transplantation, though the pathways are still largely not understood. T cells that contribute to this process include CD8+ and CD4+, which all have different roles depending on what subtype they differentiate into, as that will largely determine the cytokine they will be producing [89,90]. Some murine studies have shown that transplantation is associated with increased survival rates and along with a decrease in T cell counts [91]. Not all is grim and inflammatory with respect to these responses. During the recovery period after IRI in transplantation, Treg cells can mitigate fibrosis and injury [92]. 

### 2.5. Apoptosis in Cancer and IRI

Apoptosis, or programmed cell death, normally happens in living organisms during cell turnover of tissues, elimination of damaged or infected cells, development, and aging. Conversely, for cancer cells, apoptosis is a process that must be escaped if they are to survive and continue proliferating.

Caspases play a central role in the apoptotic cascade, and their activation can come about via two main pathways [93]. The first one, known as the extrinsic pathway, is triggered by signals from outside of the cell via either natural killer cells or cytotoxic (CD8+) T cells. Key players in this pathway are transmembrane proteins belonging to the tumor necrosis factor family: the tumor necrosis factor-alpha (TNFα) and the Fas ligand (FasL), expressed on effector cells. Their receptors on target cells are the type 1 TNF receptor (TNFR1) and a closely related protein called Fas (CD95), respectively. The ligand-receptor binding activates downstream signaling that induces the release of caspases and ultimately leads to apoptosis. The intrinsic pathway, on the other hand, is triggered by non-receptor-mediated signals that initiate within the cell itself. It is also known as the mitochondrial pathway because the stimuli that activate caspases originate within the mitochondria [94]. The intrinsic pathway is regulated by both pro- and anti-apoptotic genes and is often initiated in response to stressful stimuli such as increase in ROS or hypoxia.

The ability to circumvent programmed cell death is a well-recognized cancer feature of malignant cells, which greatly prolongs their lifespan. Either downregulation of pro-apoptotic genes, such as p53, Bclx, Bax, and BAD, or upregulation of anti-apoptotic genes, such as IAP, Bcl-2, Bcl-xL, and Brag-1, are frequently found in cancer cells [95]. Reduction of caspase functions and/or interruption of death receptor signaling are also encountered [93].

Nutritional stress, especially glucose withdrawal, should cause cell death of proliferating cells. However, as previously discussed, an increase in glucose uptake and higher glycolysis rate make cancer cells more efficient in supplying energy sources and, thus, more resistant to apoptosis [96]. This may be due to constitutive expression of HIF-1α, AKT, or any other molecule capable of stimulating these processes. In addition, significant reprogramming of further metabolic pathways has been observed in cancer cells, including Kreb’s cycle, pentose phosphate shunt, metabolism of essential and non-essential amino acids, and of lipids [97,98]. On the whole, such intricate interplay between oncogenic signaling, biosynthetic routes, and utilization of energy substrates enable tumor cells to survive and grow in a microenvironment in which a normal cell would go into quiescence, senescence, or death.

Apoptosis is common postoperatively in organ transplantation due to IRI. The acute rejection usually occurs at the site of the epithelium in the renal tubular system and rapidly leads to tubular atrophy. Meanwhile, chronic renal allograft rejection happens in a gradual fashion due to constant, non-reversible damage [99]. Cell metabolism correlates closely with perfusion; therefore, in IRI, metabolism becomes deranged and can lead to alterations in the electron transport chain as well as increased levels of ROS. When damage remains below a certain threshold, the renal tubules are able to repair the damage, mitigating the loss of functioning parenchyma and glomerular filtration ability [100]. Therefore, as essential as reperfusion is for cell survival, it is this same reperfusion that can also accelerate the apoptotic pathways in IRI. Mitochondrial damage is the ultimate deciding factor of apoptosis in IRI and has been shown in murine models. Evidence supporting IRI-related apoptosis has been published, attributing this apoptosis to mitochondrial dysfunction and caspase activation in a likely fashion as the one seen in cancer cell metabolism. Additionally, this caspase activation correlated to the ischemic period and the level of apoptosis in the period of reperfusion [101]. 

### 2.6. Mitochondrial Function in Cancer and IRI

Mitochondrial function is a topic of importance when discussing malignancies and their cellular metabolism. Mitochondria are responsible for energy provision, maintenance of the redox status, generation of ROS, control of cytosolic calcium (Ca^2+^) levels, production of acetyl-CoA and pyrimidines, and initiation of apoptosis [102]. There are two signaling pathways that modulate the structure and functions of mitochondria: anterograde signaling and retrograde signaling. The first one proceeds from the nucleus to the mitochondria through transcription of nuclear genes and cytoplasmic synthesis of proteins, which regulate mitochondrial biogenesis and activities. The second one goes in the opposite direction, from mitochondria to the nucleus, and is activated by low mitochondrial DNA (mtDNA) copy number, mtDNA mutations, alterations of the electron transport chain, or genomic mutations detrimental to mitochondrial functions [103]. 

It is known that many cancers tend to preserve their mtDNA as well as an intact electron transport chain. Fully functional mitochondria allow for metabolic processes such as respiration to occur properly and supply the cancer cell with ATP. In fact, there is evidence that a disruption in mitochondrial function can decrease tumorigenesis, and defective mitochondria which do not generate ROS were shown to hinder Kras-induced anchorage-independent growth of cancer cells [60].

On the other hand, mtDNA mutations, as well as genomic mutations involved in mitochondrial metabolism, are not uncommon in tumors, whose effect is to alter mitochondrial activities and foster tumorigenesis by enhancing cancer cell plasticity in adverse environments. Alterations of mitochondrial metabolism that increase ROS production stimulate cancer cell proliferation, as discussed below. A drop in the mitochondrial membrane potential or mutations of promyelocytic leukemia (PML) gene reduce mitochondrial Ca^2+^ uptake, thus increasing the cytosolic Ca^2+^ concentration. This ionic imbalance makes activation of the mitochondrial intrinsic apoptosis pathway more difficult and also triggers mitochondrial retrograde signaling that prompts the upregulation of genes implicated in tumorigenesis and tissue invasiveness [78]. It was observed that when malignant cells lacked mtDNA they resulted in delayed tumor growth, but these cells were able to recover respiratory function by acquiring mtDNA from host cells, after which they became more efficient in tumor formation [67]. This further supports the idea that mitochondrial function is essential for tumorigenesis and that it can be molded over time and in different conditions on the particular needs of cancer cells.

Cellular ROS mainly come from mitochondria and NADPH oxidase. Mitochondria use oxygen to generate energy in the form of ATP. They aggravate ATP depletion during IRI and increase the production of ROS in reperfusion. Excessive mitochondrial edema has been proven to hinder mitochondrial function and contribute to the progression of disease and IRI [82]. Additionally, the mitochondria are involved in the metabolism of heme and iron sulfur, which will induce further ROS production [83].

### 2.7. Production of Reactive Oxygen Species (ROS) in Cancer and IRI

A change in redox states has been involved in various diseases, and the role of ROS within cancer development and progression has been of increased interest over the years, yielding many bodies of work [104]. Chio et al. mention the work on vitamin C use in terminal cancer patients by Linus Pauling as a catalyst for interest in this field itself. Added to his work, Cameron’s work was also of great importance in the emergence of Vitamin C therapy for cancer cell patients. He described that high doses of Ascorbic Acid in patients with advanced cancer had benefits in the form of symptom relief and survival. [105] There are additional publications where the roles of ROS, vitamin C, and cancer are discussed. Fritz et al. mention the mechanism of high dose ascorbic acid being a generation of ROS, in the form of hydrogen peroxide, that becomes intolerable to the cancer cell as it is not able to metabolize it due to tumor cells lacking catalase [106]. However, the fall of this hypothesis was swift and tough when the Mayo Clinic performed a similar study and did not obtain similar results. This caused the interest in this therapeutic approach to decrease greatly [107]. 

**Figure 6:**
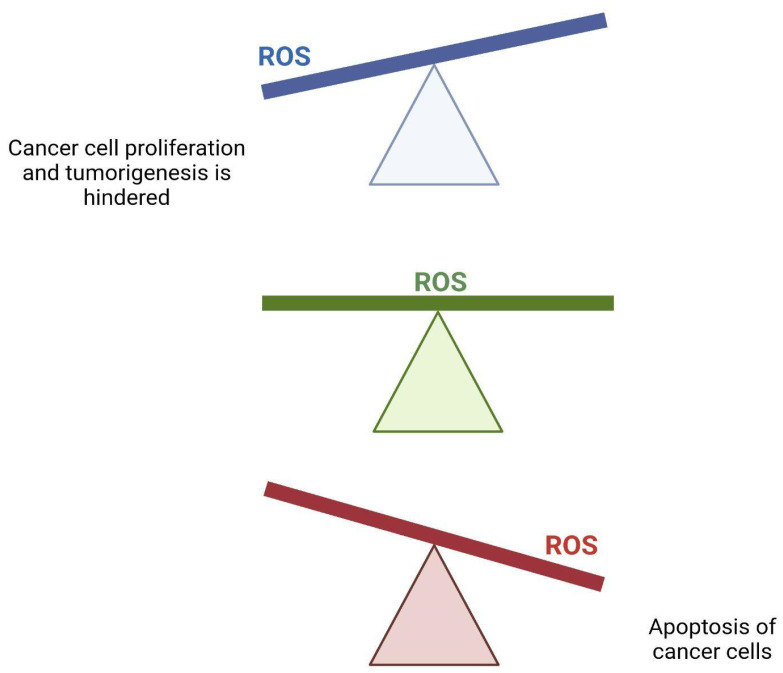
Effect of ROS on cancer cells. A homeostatic balance of reactive oxygen species (ROS) is necessary for cancer cells to survive and thrive. A lack of ROS will hinder proliferation and tumorigenesis, while an excessive amount of ROS will trigger apoptosis.

The alterations we see in metabolic processes can increase the rate of production of ROS. [12] ROS can be both beneficial and detrimental to cancer cells (Figure 6). Its role can be highly dependent on the concentration of ROS in each tumor cell and has important signaling capabilities. ROS has been thought to play a role in initiating both the progression and metastasis of cancer. Murine models have demonstrated that both metabolic metabolism and the generation of ROS are indispensable in cell proliferation and tumorigenesis [108]. On the contrary, high levels of ROS are linked to apoptosis via an antitumorigenic signaling pathway that will then induce oxidative stress that can damage and kill cancer cells. This is a mechanism associated with some chemotherapy agents [109]. In order to favor the pathways that predispose to tumorigenesis, cancer cells must maintain a homeostatic balance with regard to ROS to prevent ROS increases to the point of cell death [110]. Cancer cell ROS production is able to inactivate oncogenic membrane protein caveolin 1 in surrounding stromal fibroblasts. This will slightly reduce mitochondrial function and will increase the production of lactate in these fibroblasts. Secreted stromal cell lactate then fuels cancer cell oxidative metabolism, which drives tumor growth and proliferation. This is also known as the reverse Warburg effect [111].

Ischemia, also known as tissue hypoperfusion, indicates the deficiency in oxygen-carrying arterial blood supply to tissues. Oxygen depletion could potentially lead to a collapse of the electron transport chain in mitochondria, a fundamental component for oxidative phosphorylation and aerobic respiration in order to generate ATP, the molecular coin of cells. ATP depletion is often associated with detrimental effects on intracellular processes, including those of transcriptional, catabolic, transport, and synthetic nature, which ultimately affects cellular viability. More specifically, this includes clumping of nuclear chromatin, ribosome detachment leading to impaired protein synthesis, and dysfunction of ATP-driven pumps, such as membrane sodium-potassium pumps (Na^+^/K^+^ ATPases) and reuptake of Ca^2+^ ions by calcium pumps (Ca^2+^ ATPases) on the endoplasmic reticulum and intracellular enzymatic reactions [3,112].

The automatic solution to ischemia was thought to be prompt reperfusion, although oxygen reintroduction can initiate the formation of ROS, which drives further oxidative stress. Multiple events underpinning IRI, namely the generation of free radicals such as ROS in parallel to the decrease in antioxidative agents, result in increased oxidative stress. Altogether damage to DNA by ROS and oxidative stress can activate downstream pro-inflammatory cytokine cascades, which ultimately leads to cellular damage with loss of graft function and acute rejection [113].

**Figure 7:**
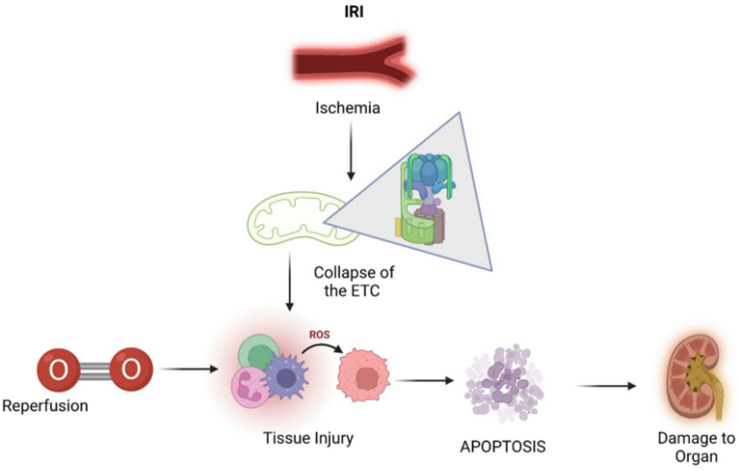
IRI leading to apoptosis and subsequent organ damage via production of ROS. (IRI = ischemia–reperfusion injury, ROS = reactive oxygen species.)

Additionally, there are other biochemical events occurring during ischemia that do not contribute to the ischemic injury immediately but that subsequently trigger a cascade of injurious events at the time of reoxygenation with blood reperfusion and, in this way, exacerbate the previous tissue injury. More in detail, during the time of reperfusion, the restoration of blood supply influences the oxygen structure, with the captation of a single electron; this produces the superoxide anion, the first ROS [114]. This oxidative stress resulting from the related hypoxia ROS production could then lead to apoptosis or, in alternative, if the damage is sustained but not lethal, renal tubules possess the capacity to repair, mainly in relation to the existing antioxidant systems to eliminate the reactive intermediates or are exhausted because of the intrinsic quality of the organ, the functioning parenchyma is replaced by fibrotic tissue, with progression towards chronic deficiency and loss of filtration capacity (Figure 7).

Among the other possible ROS originators, mainly oxidoreductases, particular attention should finally be given to NADH and NADPH oxidase. Interestingly, dissimilar to other enzymes that give rise to ROS secondary to their specific catalytic process, NADPH oxidase is, in fact, the only enzyme whose primary function is to produce ROS [115], and specifically for the kidney, the primary origin of ROS in the renal cortex is the NADPH oxidase itself [116]. 

## 3. Conclusions

Cell metabolism plays a crucial role in all disease pathophysiology. Given the importance, a deeper understanding of cellular bioenergetics brings new knowledge into two major issues of the current era: cancer defeat and reconditioning for replacement of an end-stage organ failure. Although many issues still require attention, the potentiality to repair what has been altered by targeting biosensors, any organelle or subcellular compartment of interest can potentially unveil new horizons for future research, for example, to constitute the skeleton for organoids or to administer a molecule able to kill only tumor cells. To further elucidate compartmentalized cellular metabolism and explain more fully mechanisms such as glycolysis, ROS production, and neoangiogenesis appears fundamental, as many of the most important biological questions have been rephrased as chemical problems.

As a general summary, in cancer cell metabolism, we see metabolic pathway alterations that support tumorigenesis. These include aerobic glycolysis, neogenesis, evasion of the immune system, evasion of apoptosis, mitochondrial function remains intact, and a homeostatic balance of ROS, whereas in IRI, we see changes that contribute to tissue damage. These include anaerobic glycolysis, neogenesis, a heightened immune response, apoptosis, mitochondrial dysfunction, and an increase in ROS production.

In addition, as illustrated in the current review, cellular metabolism integrates a complex set of biochemical pathways committed to the preservation of cellular functions, including the production of energy by the degradation of chemical compounds and the construction of cellular structures using precursors, but some metabolic processes are dispensable or not essential for the short-term survival of cells, constituting the secondary metabolism and potentially then being utilized for selective target actions. Importantly, for example, the insights and understanding behind the underlying molecular mechanisms of IRI are important for the identification of suitable biomarkers and the development of different novel reconditioning approaches such as small interfering RNA (siRNA), drug delivery for attenuating pro-apoptotic molecules, and downstream signaling pathways. Altogether, these interventions could be adopted during organ preservation, to counteract damage due to IRI and reduce the risk of post-transplant complications. Additionally, continued research in cancer can be applicable to IRI and vice versa, advancing the understanding and treatment modalities used for both.

We hope to have provided most general view considering main metabolic pathways and the relative products as an essential part of the biochemical cell processes and disease pathophysiology since the future of medicine depends on the primary metabolism for supplying the enzymes, energy, and substrates with a specifically tailored approach to cure the disease.

## Abbreviations

APC	Antigen-presenting cell
AKT	Serine/threonine kinase 1
ATP	Adenosine triphosphate
CAFs	Cancer-associated fibroblasts
CIT	Cold ischemia time
DBD	Donation after brain death
DCD	Donation after circulatory death
DGF	Delayed graft function
ECD	Extended criteria donor
ECM	Extracellular matrix
EGF	Epidermal growth factor
EVNP	Ex vivo normothermic perfusion
FADH2	Flavin adenine dinucleotide
FasL	Fas ligand
FGF	Fibroblast growth factor
GLUT-1	Glucose transporter 1
GF	Growth factors
HIF	Hypoxia-inducible factor
IRI	Ischemia-reperfusion injury
MCT	Monocarboxylate transporter
mtDNA	Mitochondrial DNA
MHC	Major histocompatibility complex
mTOR	Mammalian target of rapamycin
NADH	Nicotinamide adenine dinucleotide hydride
NADPH	Nicotinamide adenine dinucleotide phosphate
OPS	Organ preservation solutions
PDGF	Platelet-derived growth factor
PI3K	Phosphatidylinositol-3-Kinase
PTEN	Phosphatase and tensin homolog
ROS	Reactive oxygen species
siRNA	Small interfering RNA
TAMs	Tumor-associated macrophages
TGF-β	Transforming growth factor-beta
TME	Tumor micro-environment
TNF	Tumor necrosis factor
TSC	Tuberous sclerosis complex
TSP-1	Thrombospondin-1
VEGF	Vascular endothelial growth factor
VHL	Von Hippel-Lindau
WIT	Warm ischemia time
